# Autoethnography and severe perineal trauma—an unexpected journey from disembodiment to embodiment

**DOI:** 10.1186/s12905-015-0249-3

**Published:** 2015-10-21

**Authors:** Holly S. Priddis

**Affiliations:** School of Nursing and Midwifery, College of Health and Science, University of Western Sydney, Building EB, Parramatta Campus, Locked Bag 1797, Penrith South, DC NSW 2751 Australia

**Keywords:** Autoethnography, Severe perineal trauma, Mixed methods, Qualitative, Postnatal morbidities

## Abstract

**Background:**

There is a lack of research reporting on the physical and emotional experiences of women who sustain severe perineal trauma (third and fourth degree tears). When the researcher identifies with the group being researched, autoethnography can allow an insight into the experiences of the marginalised group through the telling of a personal story. The aim of this paper is to share the journey travelled by an autoethnographer who on examining the issue of severe perineal trauma came to understand the challenges and rewards she experienced through this reflective and analytic process.

**Methods:**

A transformative emancipatory approach guided the design, data collection and analysis of findings from this study. For this paper, a multivocal narrative approach was taken in presenting the findings, which incorporated the words of both the autoethnographer and the twelve women who were interviewed as a component of the study, all of whom had sustained severe perineal trauma.

**Results:**

As an autoethnographer, being a member of the group being researched, can be confronting as the necessary reflection upon one’s personal journey may lead to feelings of vulnerability, sadness, and emotional pain. The transformation from disembodied to embodied self, resulted in a physical and emotional breakdown that occurred for this autoethnographer.

**Conclusion:**

Autoethnographers may experience unexpected emotional and physical challenges as they reflect upon their experiences and research the experiences of others. When incorporating a transformative emancipatory framework, the hardships are somewhat balanced by the rewards of witnessing ‘self-transformation’ as a result of the research.

## Background

Perineal trauma occurs during vaginal birth when injury is sustained to the perineum (the area between the vagina and the anal sphincter). Severe perineal trauma (SPT) is when injury occurs to the perineum with trauma extending to the anal sphincter complex (third degree tear); or a fourth degree tear, this involves injury to the perineum involving the external, internal and epithelium of the anal sphincter [1]. The majority of research on the topic of SPT focuses on risk factors and causes, and the methods of repair to minimise short and long term morbidities [1–3]. Morbidities that can occur following SPT are as a result of damage to the surrounding nerves, disruption of the anal sphincter complex, and extensive scarring of the perineum and anal sphincter. Following SPT, some women remain asymptomatic, whilst other women may experience symptoms including urinary, flatus and faecal incontinence, haemorrhoids, and dyspareunia [4–6].

In addition to the physical morbidities that women experience as a result of SPT, there is evidence that women may face multiple psychosocial challenges as a consequence of this birth related trauma [7–9]. Studies report experiences of social isolation and marginalisation due to ongoing symptomatology, particularly those symptoms including perineal pain, urinary and faecal incontinence [10]. Previous research has suggested that SPT may also alter a woman’s understanding of her identity as a sexual being, as morbidities may affect her ability to engage in sexual activities and as a result impact upon her relationship with her significant other, and her sense of self [7, 8, 10]. However, despite the impact that SPT has on the physical and emotional wellbeing of women, little research has been undertaken exploring the experiences for women who have sustained SPT. This is concerning given SPT is an increasing reason for litigation in maternity care [11, 12].

I undertook a mixed methods study with the aim to understand the experiences of women who have sustained SPT using a transformative emancipatory framework. The motivation for the research that I undertook for my doctoral research project was health services transformation. My goal was to bring about change in health service provision and care for women who have sustained severe perineal trauma (3rd or 4th degree tears) during childbirth. There were four stages to the mixed methods study: A meta-ethnographic analysis of four qualitative papers reporting on the experiences for women who had sustained a physical postpartum morbidity including SPT [7]; a linked data study which reported upon the risk of recurrence, related morbidities and subsequent mode of birth for women who experienced SPT in NSW between June 2000 and July 2008 [13]; in-depth interviews to explore the experiences of women who had sustained SPT [14]; and current service provision in New South Wales from the perspective of Clinical Midwifery Consultants and women [15]. The assumption is that producing evidence and making this available will result in health service transformation.

### An autoethnographic approach

I took an autoethnographic approach to this study as I had my own experience of a 4th degree tear following the birth of my second baby, and the resulting long term consequences have had a significant impact on my physical and emotional wellbeing. I hoped that by acknowledging the journey of women, and through disseminating my work, I would inspire reflection in health professionals to create change within themselves and the health services within which they work. It has been proposed that a transformative-emancipatory framework is a way of developing a critical investigative research design and advocating for change and challenge social injustices through the research process for marginalised groups [16, 17]. It is further influenced by the theoretical lens through which the research is viewed. In this mixed methods study an interpretive feminist lens was used which allowed for the exploration of gender related oppression and marginalisation, valuing the voice of women who described and shared their lived experience of SPT [14, 18]. Further, when designing the methods of data collection, consideration should be given to ensuring that the chosen design and analysis process will result in transformation through social change [17, 19, 20]. In a transformative paradigm, participation of members of the group is valued, to ensure the findings of the research reflect the needs of the community.

When a transformative-emancipatory paradigm is used, the researcher may be a member of the marginalised group as a result of illness, circumstance or from birth. Through my personal experience of SPT I was able to self-identify with the marginalised group under investigation, therefore, I chose to take an autoethnographic approach.

Autoethnography arises from a combination of an autobiographical approach and ethnographic methodology, which focusses specifically on reflexivity and self-consciousness [21, 22]. When incorporated into a research project, the experiences of the autoethnographer are considered vital to the data in understanding the marginalised group of which they are a part [21]. Ellis ([23], p. 10) describes autoethnography as not only a way of knowing about the world, but “has become a way of being in the world, one that requires living consciously, emotionally, and reflexively”, and that as autoethnographers we must “…observe ourselves observing, that we interrogate what we think and believe…”.

During the conduct of this research and through the process of using an autoethnographic perspective, I did not anticipate that this would result in transformation within myself. I had expected that the research would present personal challenges as I reflected upon my own experience, but I was overwhelmed and unprepared for how confronting this reflection process would be; how emotionally—and consequently—physically challenging this journey would be. Therefore this paper explores the journey of the autoethnographer within this study and the insights gained into this important area of women’s health. It further explores the harrowing personal toll, and soul searching, that eventuated along the way for this researcher as a consequence of using an autoethnographic approach. The paper is presented as a multivocal narrative [24], as woven throughout my words and personal reflections are the stories of the women who participated in this research project. This identifies contrasts and similarities between my story and the stories of other women who have sustained SPT. The quotes that are used are directly from the interviews with the women, those that have been published previously are cited to that publication. Pseudonyms are used to protect the identity of the women.

## Methods

The growth of autoethnography as a method conflicts with traditionalist perspectives which value objectivity, validity and reliability in the design and analysis of research. As a result there are multiple definitions and presentations of this methodological approach [22, 25]. Evolving from the work of ethnography, whereby the researcher explores meaning behind behaviour and interactions in a specific cultural group within a natural context, autoethnographers identify their position within the cultural group being researched and weave their personal experience into the analysis and interpretation of the collected data [16, 26, 27]. There are two key autoethnographic approaches in research design, analysis and presentation—evocative or emotive, and analytic.

### Two autoethnographic approaches

The origins of autoethnography draw upon emotion, an autobiographical presentation of the journey of the researcher, and are therefore described as evocative [25, 28]. Evocative, also described as emotive autoethnography values the story of the autoethnographer, however it “transcends mere narration of self to engage in cultural analysis and interpretation” [29]. Through a reflective writing process, autoethnographers make themselves vulnerable as they share their own story to benefit the group they are researching. As stated by Denzin ([30], p. 228), autoethnographers “…bypass the representational problem by invoking the epistemology of emotion, moving the reader to feel the feelings of the other.”.

When undertaking analytic autoethnography, Anderson [21] suggests that there are five key features: complete member research status, analytic reflexivity, narrative visibility of the researcher’s self, dialogue with informants beyond the self, and commitment to theoretical analysis. Analytic reflexivity requires the researcher to have an awareness of their place within the research and the potential impact this has upon the interaction with study participants and interpretation of data [31]. The use of analytic reflexivity attempts to ensure objectivity and authenticity throughout the research process [21, 32]. However this does not sit well with those who value evocative autoethnography, with Ellis and Bochner stating ([22], p. 440): “If you turn a story told into a story analysed…you sacrifice the story at the altar of traditional sociological rigor. You transform the story into another language, the language of generalization and analysis, and thus you lose the very qualities that make a story a story”.

In response to the division between the emotive and analytic approaches of autoethnography, Tedlock [33] presents examples of autoethnographic literature where authors have chosen to interweave both emotive and analytic approaches in writing. In the work presented by Giorgio, she demonstrates this interweaving of both approaches through her reflection: “When I sit down to write, I find the story behind the memories; I then begin to make sense of those memories, their meaning for me and for others” ([34], p. 406). As a novice autoethnographer I valued both the emotive and analytic perspectives, and felt it necessary to combine both approaches not only to truly embrace and travel the bumpy road that is the autoethnographic journey, but to fulfil doctoral research requirements.

### Positioning myself amongst the participants

In this study, while I am considered a member of this marginalised population, I acknowledged that the experiences of the women who have sustained SPT are also individual; therefore my position as complete member researcher provides only one perspective on exploring the experience of the marginalised group [31]. In depth interviews were conducted with 12 women who had sustained SPT, with the purpose of the interviews to explore the way women understand and have experienced SPT. This included their experiences of, and interactions with, health services. The recruitment, participation process, demographics of participants, and findings have been previously published [35]. To ensure informed consent, participants were provided with an information sheet prior to the interview being conducted. Participants were then asked to read and sign the consent form if they agreed to participate. During the interview process, I provided full disclosure of my experience of a fourth degree perineal tear and associated morbidities. However, when a woman became distressed during the interview, it felt inappropriate to tell my story as I was concerned that this would shift the focus from the woman to myself as the researcher. In this situation, I made the woman aware of my story at a more appropriate time following the interview. Ethics approval was obtained by Western Sydney University Human Research Ethics Committee.

This disclosure immediately allowed for identification as an “insider” and facilitated a more open discussion. The role of insider occurs when the researcher has dual identities as both researcher, and as a member of the marginalised group being researched [36, 37]. During a few of the interviews an interesting shift occurred following disclosure of my experience. The women—as a result of identification with my experience—revised roles and became the interviewer. The women were curious as to my daily management strategies, recovery following treatments and surgeries, with a particular focus on sexuality and the practical aspects of intercourse with my partner. I let this transference of ownership of the interview evolve and followed the path set by the woman, to see where the journey would end.

The autoethnographer, as a result of critical self-reflection, may experience a change in perspective as a result of the research itself [17, 38]. This is further explored by Schwalbe ([39], p.58) who describes the impact of autoethnography on the researcher: “Every insight was both a doorway and a mirror—a way to see into their experience and a way to look back at mine.”. In reflecting on the roots of autoethnography, Bochner described how autoethnography found strength as an alternative to the limitations found in the social sciences, and “feeds a hunger for details, meaning and peace of mind” [40]. The growth of this qualitative genre has allowed for personalised interpretations and explorations of experience, “peace of mind” is not however, something that is always experienced by the autoethnographer, and this certainly rings true for my own experience which is explored in this paper [41, 42].

## My story

### My first baby

My first baby arrived when I when I was 19. Now, 20 years later, I am unable to recall every part of my son’s labour and birth. I remember feeling overwhelmed and alone, even though my family were in the room, and requesting pain relief. I remember pushing on my left side, one midwife holding my leg in the air and the other midwife saying “You are tearing, we need to cut you”. I didn’t feel the episiotomy; following this my son was born, not breathing. I remember bells and alarms and a resuscitation, and after a scary amount of time he was brought to me, my 7 lb 1 oz wrinkly little boy. And then I remember the suturing by a doctor I did not know. It was so incredibly painful, and frightening. And even with the sleepless nights, the cracked nipples and mastitis, what I most vividly recall is the suturing.

The women in this study described that feelings such as vulnerability, discomfort and fear were directly related to the way in which they were cared for by their midwife, obstetrician, and the health professional undertaking the repair. Women described these interactions as often inappropriate, recalling the facial expressions and the way health professionals often did not communicate directly to the women but discussed her perineum between themselves:

*“And [the doctor] didn’t really want to talk while she was suturing, she just had this disgusted look on her face when she was doing it. It was horrible, it wasn’t nice…” (Ava)* ([35], p. 4)*.*

### My second baby

When I became pregnant with baby number two, 5 years later, those memories were still fresh and raw. It was important to me that those experiences weren’t repeated, that my baby was born safely, that my body was supported to give birth without trauma. I found a midwife in a birth centre that I connected with, who provided the care that I needed. I carry her name forever in my heart, and she ultimately became one of the core reasons I became a midwife. Following a long labour when I reached second stage, I had an overwhelming urge to push and an instant relief. My 9 lb 5 oz daughter was born following a 9 min second stage.

I didn’t feel the tear. After some skin to skin, attempts at breastfeeding and bleeding that would not settle, my midwife examined me and looked concerned. She bought a doctor in who did a second examination and then explained to me that I had a 4th degree tear, down to and through my anal sphincter. I was shocked. I was then taken to a treatment room. I left my new daughter with my husband, and multiple injections of lignocaine and excruciating pain later, had the fourth degree tear of my perineum sutured. What got me through this experience was the most beautiful student midwife who came in with me, held my hand, and murmured comforting words to me while I cried in pain.

Women may experience fear around the anticipation of pain during the suturing process, the damage sustained to their perineum, and that they may be sutured incorrectly resulting in further perineal damage [7, 43]. For some women, they described how they also experienced pain during the process of perineal assessment and suturing:

*“…there was a doctor that came and had a look. She was quite rough, I thought. She was really poking and shoving gauze in there. I was screaming my head off. It was really awful.” (Indie)*

I went back to the hospital two weeks after my daughter was born. I remember discussing my breastfeeding issues, but nothing else. I had my 6 week check up with my GP, who read my notes, put his finger into my anus—remarked that it felt fine, and I was sent home.

In this study, women described the value of receiving comprehensive information and compassionate care following the birth, however these appear to be lacking in the current system [7, 35].

*“I think [the doctor] could have definitely told me who to call if I had any problems. I wasn’t given any numbers or information or anything. Maybe just some recovery tips, or I think they could have told me what happened. I think the system let me down, I think it did. I’d like to think that other women who go through this have a lot more support…” (Lola).*

### My leaking body

Three months after my daughters’ birth I had a dentist appointment. I remember parking the car, going up the escalator into the shopping centre where the dental rooms were. I was walking along when I felt something run down my legs. I quickly ran to the toilet, thinking perhaps I had gotten my first period. But I was horrified to see that I had become incontinent for faeces. I remember sitting in the toilet, crying, wondering what on earth to do. I threw my underpants out, and I tried to clean myself, luckily I had nappy wipes in my handbag and I used those to clean myself the best I could. Once I composed myself I went into a chemist and used one of their sample perfumes to spray myself, I was completely paranoid that I smelt of faeces. I still had to go to the dentist appointment but the whole time I lay there praying that I didn’t smell and that no more would run out. I have never forgotten that day, or the absolute disgust that I felt with myself that I had no bowel control.

‘A leaking body’ is described in the literature as immature [44, 45]; this link was reflected in the words of women who described uncontrollable bodily functions, particularly faecal incontinence, as being dirty like a toddler, or a naughty child [35, 46]. Studies also report the efforts women make to conceal body functions, including menstruation, urinary and faecal incontinence, to conform to cultural and societal norms [47].

*“…part of me thought ‘[my best friend] will judge me if I tell her that I’m poohing my pants’. Not that I think she would’ve thought any less of me, I’m sure there would have been sympathy, but I thought it was disgusting so I didn’t want anybody else to judge me for that…” (Matilda)*

Over time I developed strategies for dealing with the episodes of faecal incontinence. I always had spare underpants and nappy wipes in the baby bag that I carried with me. I drank minimal amounts of water, I found being constipated an easy way to manage bowel control. I saw my GP but he just suggested doing more pelvic floor exercises, but with minimal control over my pelvic floor I struggled to do more than two at any given time.

In my study women described how they were required to manage their daily living activities, such as sporting activities and recreational outings, as they risked experiencing an unexpected episode of urinary or faecal incontinence. Management strategies include avoidance, or limiting activities and outings to places where there were easily accessibly toilet facilities [7, 10, 48]. Further management strategies that women have reported include the style of underwear and clothing that they were required to wear to camouflage the use of incontinence pads, and to avoid further irritation of the perineum and associated pain [7, 10].

*“Time went on and I just kind of changed a few things, like stopped wearing G-strings and carried wipes and spare undies around. I just adapted my lifestyle to it.” (Lola)* [35]

### More babies and more symptoms

Over the next two and a half years I had two more children by caesarean section. Each pregnancy made my symptoms worse. Throughout my fourth pregnancy my perineum felt heavy, weak and the inability to complete bowel movements occurred with every trip to the toilet due to a lack of control that remained of the anal sphincter muscles. Now a mother of four, I was becoming nervous about leaving the house following the memory of the trip to the dentist all those year ago, and the fear of being incontinent. But I put up with it. I looked after my babies, commenced my midwifery training, and did all the things mothers do. After attempts at repairing the damage through surgery, then developing a temporary fistula as a co-morbidity, to this day 14 years after my fourth degree tear, I have ongoing incontinence of flatus, and difficulty holding onto faeces for any length of time. I experience pain in my perineum and anal sphincter.

## The need to do this study

It is perhaps not surprising then that I wanted to explore this issue for other women in my own part of Australia where I live for my PhD. Reading about autoethnography as a methodological approach made me realise that while potentially painful, this would provide an additional depth to the analysis of the data. As Allen and Piercy ([49], p. 160) describe: “In that place of vulnerability, I am more open to hearing the voices of others, particularly those in marginalized positions. I am less ready to dismiss the experiences of others, or to superimpose theories that will distance myself from connecting with them.”. Autoethnography provides a platform from which I can detail and express my process of embodiment and the consequent outcomes.

## Results

### As researcher as woman as researcher

The use of autoethnography can be seen as both a strength and limitation in conducting qualitative research. Whilst being a member of the marginalised group provides an “insider” opportunity as researcher when recruiting and interviewing participants, a limitation is that the research process can be too confronting for the autoethnographer as the process of conducting research and the necessary reflection upon one’s personal journey is similarly confronting, leading to feelings of vulnerability and emotional pain [25, 28, 41]. To address the potential for bias from the authoethnographer dominating, in presenting research from an analytic autoethnographic perspective, it is important that the researcher is visible, reflexive to ensure objectivity and authenticity, and committed to theoretical analysis [21].

As I remained constantly mindful that my experiences were not those of the women, and that each story stood alone, I was often struck by the similarities of our stories. As I reflected upon the coping mechanisms adopted by the women, I - in turn, reflected upon my own. In the process of conducting this study, this level of critical self-reflection was both confronting and upsetting. This level of reflection revealed that I had moved towards a “completely different normal” by compartmentalising the perineal morbidity and associated long term symptoms. This theme was also identified by the participants in one of the studies I undertook [35]. However in order to be reflective and analytic during the process of thematic analysis, this coping process of compartmentalising needed to be pulled aside so that I could become fully immersed within the data. Viewing the damaged perineum from a mechanistic viewpoint, as a faulty object, a disembodied or component part, had been protective and safe, while the paternalistic discourse of the feminine body as weak and inferior was confronting and upsetting. Autoethnographers have explored this dualistic role of autoethnographer and self, with Olson ([50], p. 7) stating: “The dualistic role of a personal survivor and an academic is a reflexive one, each informing the other, never separate from one another”.

The relationship that I developed with the women who participated in the study, following full disclosure of my own experience, fostered a closeness that remains to this day. Many of the women have remained in contact with me to report follow up assessments and test results, subsequent babies and general wellbeing. This has been a double edged sword as although I am humbled that these women have taken me into their hearts as a confident, it has been accompanied by an emotional burden that has weighed heavily on my heart.

### Reflections: autoethnographic discoveries

My understanding of what it is to be an insider and an autoethnographer, and the enormous impact this has had on me personally is best represented by sharing my personal field notes or “reflections”. These reflections included the use of a personal online journal, and a summary of emails that were sent to my primary research supervisor as I attempted to make sense of my experience. This is one of the first reflections that I completed:

(21st November, 2011)

*Five interviews so far. I expected to find this journey challenging, however I am finding it difficult in different ways than what I expected. I thought that hearing the stories told by women would make me reflect upon my own ongoing experiences and that that would be confronting. However, what I am finding distressing is that women have experienced pain, incontinence and feelings of distress around their birthing experience....*

Within this same reflection my beginner level understanding too of what autoethnography was is apparent here:

*Threading autoethnography throughout the interviews does not always feel appropriate to me. When a woman is distressed, or has experienced extensive physical and psychological ramifications—I feel it is inappropriate to tell my story, that it takes away from the woman’s own experience.*

Three days later I had completed 8 interviews and my perspective was slowly shifting:

(24th November, 2011)

*During this time I became more aware of my own body, my inadequacies (as I perceived them) were magnified, and I was more aware than ever of my pain and the inability of my body to function as it should. Although I felt relief for the women who participated in my study who did not experience any symptoms following SPT, at times I felt a sadness, and to this day wonder why my body just would not heal as theirs had. I share their sorrow, their fear and understand why they try to dismiss these feelings as being ‘all in the mind’.*

There is then a gap of nearly a full 12 months between my recorded reflections, and the next reflection describes why:

(6th December, 2012)

*I had expected that the challenge would be in interviewing women that I would be confronted by their distress and that would be the most challenging part of my doctoral journey. Whilst I empathised and sympathised with these women, I left each interview a little saddened but mostly inspired to represent these women honestly and with respect, to advocate and instigate change.*

*One afternoon that changed. My own personal symptoms had been exacerbated as a result of stress of my parenting/academic workload, and one afternoon as I was doing preliminary coding of transcripts, I became overwhelmingly upset and cried. And cried and cried. This crying continued for weeks, into months, and I assumed that this occurred as a result of my workload and transcript coding. I took a temporary breather to refocus and explore why I had responded this way.*

### Crisis point—the process of a break down

I was given the amazing opportunity of being invited to present my research in Canada, a trip and opportunity I was extremely excited about. However, when I arrived in the first airport following the long haul flight I became extremely physically unwell with severe gastric symptoms that lasted the duration of my time in Canada. While I was still able to present my research I spent the majority of my time on, or near, a toilet. During this experience I was highly anxious and I remained unwell on the return home, and for the following months. I lost a substantial amount of weight due to chronic gastric problems, and a loss of appetite. I was restless, depressed and became increasingly withdrawn. I was unable to focus on work due to the restlessness and overall physical exhaustion due to the weight loss, and was unable to present my work at conferences at which I have been invited to speak.

Following many appointments with medical professionals, and surgery to identify the cause, it was found that I had not only been unwell as a result of a parasite when travelling, this stress had initiated a physical response, meaning I was now intolerant to gluten and lactose. This had further been the catalyst to trigger an overwhelming emotional response; I was experiencing depression and anxiety. I felt helpless and overwhelmed, in a state of panic each day. I was truly frightened by what was happening, I thought I was simply going crazy. On any given day my body was constantly “humming”—preparing me for fight or flight. This constant humming meant I need to be moving or standing at all times, trying to keep my body as busy as my brain, as it bounced from one thought to another unable to keep still or focussed. My house was spotless as I crawled around on the floor cleaning skirting boards at six in the morning!

So the anxiety then became a cycle—my body felt fearful or anxious, and my gastric system became upset. Then I became concerned that if I was out somewhere, away from a toilet, I would have a gastric episode and so then that triggered the anxiety. I admit, when I was in the darkest of places, that death looked attractive. However to clarify, I was not suicidal but merely considered the peace that would come with death as a reprieve from the chaos, whenever death happened.

### Disembodiment, embodiment and coping

In reading the stories told by the women, and undertaking my meta-ethnographic synthesis I became interested in exploring the concepts of embodiment versus disembodiment, whereby the dysfunctional component of the body is identified as being either part of the person or as a separate (disembodied) entity [7, 51, 52]. Research has explored how the separation between mind and body contributes towards the disconnection and disembodiment some women may experience at birth [43, 51]. At the moment when a woman gives birth and the baby and placenta are born, there is both a physical and emotional opening of the self to the other, a transition of the boundaries between the internal and external occurs [13, 52]. For women who sustain SPT at birth, there is a continuation of this physical trauma through the perineum that distorts the boundaries of the known body. While research suggests that childbirth is a “conclusion”, where women undergo a transition to a new self, for women who experience ongoing urinary, flatus and/or faecal leakage, the boundaries between the internal and external are permanently altered, challenging both physical and emotional closure for women [51, 52].

Through reading, and my own personal journey as I attempted to rediscover my sane self, I realised that for the past 14 years since the birth of my daughter, my coping strategy was based upon disembodiment, separating my traumatised perineum from my personal self. This allowed me to continue with my life and multiple commitments, as a doctoral candidate, mother of four children including one with a disability and running a business, without my ongoing morbidities affecting me on a personal, intimate level. The women I had interviewed also described this disembodiment I had not realised that this is what I had also done to survive.

On reflection, I recalled days when I experienced varying degrees of incontinence or pain I would describe them as my “bad bottom” days to those close to me, externalising the “bad bottom” and placing it as an object, clearly disembodying from the process.

Whilst this strategy of coping had served me well practically, a strategy I was comfortable with, that is being disembodied would not allow me to truly delve amongst the research and represent the stories of the women that I interviewed as I needed to. I had subconsciously adopted an embodied view of my severe perineal trauma and was not coping at all. I had uncovered a part of myself, a vulnerability and depression, which placed me in an uncomfortable place—confronting, dysfunctional. My ability to multi task my crazy life was lost, I jumped from one task to another trying to keep on track (unsuccessfully). I did not like the part of me that had emerged, I felt vulnerable and weak, and this conflicted directly with my role as a strong, efficient, perfectionist working mother.

### Re-emerging as the embodied/disembodied woman

I knew that I had work to do. Completing my PhD within my timeline and representing the women’s stories with honour and respect was important to me. So I started working again, on other things—I avoided the interviews for a while. All the de-identified interviews sat in a blue zip up folder, I found it difficult to be near or look at this folder which I kept in my study. Then, after time, I was able to carry the folder with me to research supervisory meetings, but was unable to open the folder, and to this day I feel physically unwell whenever I see that folder which has now been stored away. I transitioned my focus to the quantitative component of my research and reading autoethnographic literature. It was important to me to find others who had travelled a journey similar to mine to understand how they survived this process:

(23rd May, 2013)

*….and then I turned to autoethnography literature. I surrounded myself with the works of others who had travelled the autoethnographic path. And in doing this, I discovered a sense of camaraderie, a support group of researchers around the globe who too had travelled this journey and confronted their own personal demons. Their words wrapped around me and provided warmth and comfort for my saddened soul.*

The solace I found in the works of these amazing autoethnographers [23, 25, 53] not only provided me with strength to complete my work, but ignited in me a true passion for what autoethnography is. I have always been a writer but to write in this way has been such an incredible privilege for me.

### Caring for the autoethnographer

In documenting this experience it has highlighted for me the ethics of undertaking autoethnographic work, specifically caring for the self. When researchers prepare to undertake qualitative work, we go through a rigorous ethics approval process to ensure the participants are well supported and protected from any potential risk of harm. While ethical consideration is extended to the individual conducting the research, self-care strategies are more difficult to develop and put in place when the person at risk is yourself. As an autoethnographer conducting research not only do I have to be mindful to apply ethical considerations to the participants of the study, but must develop a way to protect myself, and my significant others, from risk of harm [29, 54]. Allen and Piercy ([49], p. 156) state: “By telling a story *on* ourselves, we risk exposure to our peers, subject ourselves to scrutiny and ridicule, and relinquish some of our sense of control over our own narratives.”.

## Discussion

### Learning to survive

What I have shared with you in this paper are my memories: thoughts, words and physical scars that are embedded in my consciousness. Recollection is personal, biased, and my memories are tinted with fear and sadness. The road to recovery has been difficult, but I have always been a fighter and way too stubborn for this to overwhelm me. In addition I have an amazing husband and four incredible children who have always been my light in the darkness. Through the process of researching, reading, and searching for recovery stories, I discovered helpful websites addressing anxiety which taught me how to face my anxiety each day and work alongside it to minimise my daily fear. I commenced Cognitive Behavioural Therapy along with walking kilometres each day, doing yoga, and complete dietary changes including giving up alcohol and caffeine. My research supervisors, my husband, my best friend and a few close colleagues listened to me without judgement only concern. Am I recovered? No. But I am on the path. I cannot say that these changes in my life have come from a positive place, but I can say that the changes are becoming a positive thing and I feel blessed to have been given this opportunity for self-discovery that may or may not have happened otherwise. I am healthy, fit and in tune with my thoughts and feelings. I now identify myself as a “Spiritual Adventurer”, exploring all that it is to be me. Whilst I am not yet always able to manage these overwhelming anxieties, I know that I am a strong, determined woman who has always faced her challenges, looked them straight in the eye and conquered them.

### What is the point if it does not make it better for other women?

The aim of using a transformative emancipatory research design was to allow for an in depth understanding of how women experienced and understood SPT. The study focused on the individual experiences and interactions with health care professionals to inform and advocate for change through the research process. A strength of using a transformative research approach can be demonstrated through the actions of the research participants [16, 55]. Following data collection some of the women who participated in the study initiated ongoing contact with the researcher via email and phone to inform me of their subsequent pregnancies, births and endoanal assessment outcomes. I have had the opportunity to present my research reporting on SPT, including my own experiences, at various seminars and workshops over the years. At one such seminar a physiotherapist approached and thanked me, telling me that my presentation that first day had inspired her to specialise in caring for women who had sustained SPT. She has now established a SPT clinic for women just like me.

Reflecting on the aims of my mixed methods doctoral study, one of the purposes of data collection, analysis and integration was to determine how services can be improved who provide care for women who have sustained SPT. Senior health policy makers and advisors welcomed my principal research supervisor and I into a discussion to explore how the findings of my research could be used to assist with the development of guidelines to improve and support service provision. The positive response of policy advisors and policy makers from this discussion was rewarding, as during the most difficult of times when death and the peace to be found there seemed attractive to my anxious mind, one of my many concerns was that my work had been for no purpose. To transform, by using the transformative-emancipatory approach, was my initial motivation, and to have hope that my work one day, will assist with the development of guidelines to improve health services and the care that women with SPT receive. I know now this could bring with it a sense of closure, personal transformation, and, with that, peace for me.

To witness transformation, no matter how small, is humbling. At a research presentation forum for doctoral candidates an academic staff member came up to me. She held my hands, and looked into my eyes. “You are doing such valuable work, you are a very special person. This work must have been very challenging for you, given your personal experience”. I agreed with her, describing the difficulties I faced as I was required to peel away my own coping mechanisms to immerse myself in the research. She nodded solemnly, as her eyes pricked with tears. “Twenty five years ago I gave birth. My perineum was badly damaged and the doctor said to me ‘don’t look at it, it’s all black’. And I’ve never looked, not once since that day”. She looked back up at me and gave my hands one last squeeze, “Your work is so valuable, you are very special”. She smiled at me then walked away.

## Conclusion

The journey as an autoethnographer is both a gift and a curse as the challenges that are faced by the researcher, as they reflect on their culturally constructed and protected self, can be both therapeutic and damaging. The sharing of my story with the participants not only facilitated the research, but resulted in a ‘transformation’ through disembodiment to embodiment. While my experience has not been an easy one, in preparing this paper I realised that the hardships have been eased somewhat by the opportunity to disseminate my research with the hopes of bringing about change for women who sustain SPT and postpartum morbidities. This paper goes further as it also describes and hopefully, assists, other autoethnographers grapple more safely with the notes and gifts of using this methodology.

## Abbreviations

SPT: Severe perineal trauma
NSW: New South Wales
PhD: Doctor of Philosophy
